# An Analysis of Language as a Barrier to Receiving Influenza Vaccinations among an Elderly Hispanic Population in the United States

**DOI:** 10.4061/2011/298787

**Published:** 2010-09-01

**Authors:** William S. Pearson, Guixiang Zhao, Earl S. Ford

**Affiliations:** ^1^Healthy Aging Program, Division of Adult and Community Health, Centers for Disease Control and Prevention, Atlanta, GA 30341, USA; ^2^Emerging Investigations and Analytic Methodologies Branch, Division of Adult and Community Health, Centers for Disease Control and Prevention, Atlanta, GA 30341, USA

## Abstract

*Background*. The Hispanic population in the United States is growing, and disparities in the receipt of healthcare services as a result of limited English proficiency have been demonstrated. We set out to determine if Spanish language preference was a barrier to receiving influenza vaccinations among Hispanic persons 65 years and older in the USA. *Methods*. Differences in the receipt of vaccinations by language preference were tested with both Chi-square analyses and adjusted logistic regression analyses. *Results*. Findings suggest that elderly Hispanic persons, 65 years of age and older, who prefer to communicate in Spanish instead of English, are significantly less likely to have received influenza vaccinations when compared to their Hispanic counterparts who prefer to communicate in English. *Conclusions*. Influenza infections can more often be fatal in older persons and may disparately affect minority populations such as Hispanic persons. Therefore, understanding barriers to the receipt of effective preventive health measures is necessary.

## 1. Introduction

Morbidity and mortality resulting from seasonal influenza infection continues to be a significant concern for residents of the United States, and this is especially true among the country's elderly population [1]. It is estimated that on average there are over a quarter of a million hospitalizations and approximately thirty six thousand deaths due to seasonal influenza annually [2], and that direct medical costs due to influenza infection average $10.4 billion every year [3]. Vaccinations against influenza are recommended for people who want to reduce the risk of getting influenza or transmitting it to others, and up until 2010, were specifically recommended for at-risk groups, including persons 65 years of age and older [4, 5]. Currently, all persons six months of age and older are recommended annual influenza vaccination [6]. As the U.S. population continues to age and grow, more people, especially vulnerable populations, will be at risk for developing this costly and potentially deadly disease.

Recent census data indicate that the fastest growing ethnic group in the United States is Hispanic, with a total estimated population of 43 million persons in 2005 [7]. Projections by the U.S. Census Bureau suggest that by the year 2050, the number of Hispanic persons in the United States will more than double [8]. As the Hispanic population within the United States has increased, Spanish has become the primary language of many Hispanic households in the United States [9]. In fact, in 2006, the U.S. Census Bureau estimated that the number of persons aged 5 years and older who predominantly spoke Spanish and who reportedly spoke English “less than very well,” was greater than 16 million [9]. 

The observed shift in the racial and ethnic demographics within the United States and resulting issues of acculturation have led to an increased body of research examining language preference among Hispanics in association with health care delivery and utilization. For example, one recent study examining the impact of acculturation on the receipt of genetic cancer testing among a heterogeneous Hispanic population found that language preference was a significant predictor of awareness of the genetic cancer tests [10]. More recent studies have shown that Spanish language preference can be a barrier to access and use of health care services [11, 12], and can result in less-efficient care [13, 14]. Findings from these researchers suggest that disparities in health care delivery exist because of language barriers on the patient side and also on the provider side. Therefore, non-English speaking populations may be at a higher risk for developing preventable diseases because of the observed language barrier.

Recent studies point to disparities in getting influenza vaccinations, and this disparity is clearly apparent for Hispanic persons [15–17]. Eliminating racial and ethnic health disparities in the receipt of influenza vaccinations, is a goal of Healthy People 2010; one of those specific areas being influenza vaccinations [18]. However, this goal has not yet been met. It is estimated that in 2007, vaccination coverage levels among persons aged 65 years and older were 70% for non-Hispanic whites, 58% for non-Hispanic blacks, and 54% for Hispanics [19]. It also has been estimated that eliminating these disparities would significantly reduce mortality rates among elderly Hispanic persons [20]. However, beyond the general racial and ethnic disparities previously studied, little research has been conducted regarding receipt of vaccinations based on language preference among the Hispanic population. Because of the need to reduce racial and ethnic disparities in influenza vaccine coverage, we sought to determine if Spanish language preference was a significant barrier to receiving recommended influenza vaccinations among Hispanic persons, 65 years of age and older, residing in the United States. 

## 2. Methods

Data was gathered from three years (2005–2007) of the Behavioral Risk Factor Surveillance System (BRFSS) survey. The BRFSS, collected by the Centers for Disease Control and Prevention (CDC) since 1984, is the world's largest ongoing, state-based, landline telephone survey used to collect information on health risk behaviors, preventive health practices, and access to and use of health care services primarily related to chronic conditions among adults aged 18 years and older with more than 350,000 completed questionnaires each year. The survey oversamples minority and elderly respondents and is weighted for making population estimates. The median CASRO response rate was 51.1% for 2005, 51.4% for 2006, and 50.6% for 2007. The median cooperation rate was 75.1% for 2005, 74.5% for 2006, and 72.1% for 2007 [21]. 

The analyses were limited to persons who self-identified as Hispanic, were aged 65 years or older, and who resided in one of the states that offered a Spanish version of the BRFSS. A Spanish translation of the BRFSS was completed for those states reporting that a significant proportion of residents spoke Spanish. The 27 states (AZ, AR, CA, CO, CT, FL, ID, IL, IN, IA, KS, MA, NE, ME, NV, NJ, NM, NY, NC, OK, OR, RI, TX, UT, VA, WA, and WY) that made Spanish versions of the survey available were included in this study. The language respondents chose to answer the survey (English or Spanish) was used as the main stratification variable. Sample populations for each year were 3,300 for 2005, 3,949 for 2006, and 5,125 for 2007.

Each respondent was asked two questions to determine if they had received an influenza vaccination. The first question was: “A flu shot is an influenza vaccine injected into your arm. During the past 12 months, have you had a flu shot?” The second question was: “During the past 12 months, have you had a flu vaccine that was sprayed in your nose?-The flu vaccine sprayed in the nose is also called FluMist.” However, only responses to the first question regarding a flu shot were used for this analysis since FluMist is not available for persons over the age of 49. Only “Yes” or “No” responses were used in the analyses. Responses of “Do not know/Not sure” were not used. Demographic characteristics of the population (e.g., age of the respondent, gender) were collected and used for descriptive purposes and as covariates in logistic regression models. Socioeconomic factors used included level of education, access to health care coverage, and if the respondent had one person that they considered to be their personal health care provider. For purposes of these analyses, education was operationalized as having at least completed high school or greater as compared to those who had not completed high school. Access to health care coverage was determined through the question: “Do you have any kind of health care coverage, including health insurance, prepaid plans such as HMOs, or government plans such as Medicare?” Access to a personal health care provider was determined through the question: “Do you have one person you think of as your personal doctor or health care provider?”

### 2.1. Statistical Analyses

Population estimates, with 95% confidence intervals, were made for all demographic characteristics and included whether or not the respondent had received the influenza vaccination, then were stratified by year and language preference. Differences in the receipt of vaccinations by language preference were tested for each year by using Chi-square analyses and tested at an *α* = 0.05. Differences were further tested through adjusted logistic regression analyses where the demographic and socioeconomic variables access to care and a personal health care provider were included as co-variates in the models. The dependent variable in these models was a dichotomous outcome for receiving the vaccination, or not. The main independent variable was language preference (i.e., Spanish or English) where English preference was the referent group. Odds ratios were determined for respondents choosing Spanish. All analyses were conducted in SUDAAN [22] to account for the complex sampling design of the survey. 

## 3. Results

The stratified populations of respondents choosing English or Spanish were shown to be homogenous in most demographic measurements across all three years, with three exceptions. First, in 2005, respondents with a preference for English had a significantly greater proportion of persons reporting that they had one person that they considered their personal health care provider compared to those choosing Spanish (82.2% for English preference and 73.0% for Spanish preference, *P* < .01). Second, in 2006, respondents choosing English had a significantly greater proportion of persons reporting that they had any health care coverage compared to those choosing Spanish (97.8% for English preference and 93.3% for Spanish preference, *P* < .01). Third, from 2005 through 2007, respondents choosing English had a significantly greater proportion of persons reporting that they had completed high school or greater, as compared to respondents choosing Spanish (67.2% for English preference and 32.4% for Spanish preference, *P* < .01, in 2005; 67.9% for English preference and 35.3% for Spanish preference, *P* < .01, in 2006; and 72.9% for English preference and 37.6% for Spanish preference, *P* < .01, in 2007) (Table 1).

Significant differences for the estimates of influenza vaccinations received based on language preference were found between respondents for each of the three years analyzed. For all three years, respondents choosing Spanish had significantly lower vaccination rates as compared to those choosing English (*P* < .01) (Table 2). 

Vaccination rates increased for those choosing to complete the survey in English and remained flat among those choosing to complete the survey in Spanish.

After controlling for age, sex, education, access to health care coverage, and a personal health care provider, Hispanic survey respondents choosing Spanish were significantly less likely to have had an influenza vaccination within the past twelve months compared to those choosing English (Table 3). In 2005, respondents choosing Spanish were more than 30% less likely to have received the vaccination compared to those choosing English (0.67 OR, 0.48–0.96 95% CI). In 2006, the odds of vaccination decreased to nearly half as compared to those choosing English (OR = 0.53, 95%  CI = 0.38–0.7), and in 2007 the odds fell even further to 50% less (OR = 0.50, 95%  CI = 0.38–0.65) (Table 3). 

## 4. Discussion

Using three years of recently available state-based BRFSS data, our findings suggest that Hispanic persons, 65 years of age and older, in the United States who prefer to communicate in Spanish are significantly less likely to have received their recommended influenza vaccinations as compared to their elderly Hispanic counterparts who prefer to communicate in English. Furthermore, there was no evidence that this gap narrowed during the three-year period. It appears that language preference among a Hispanic population, 65 years of age and older, in the U.S. are associated with lower rates of influenza vaccinations, indicating a possible health disparity that could have significant public health implications for the nation.

Our data on differences in the receipt of vaccinations among Hispanic persons based on language preference is similar to previous reports that examined this association. For example, using data from the 1996–1997 Community Tracking Survey, Fiscella and colleagues demonstrated that Spanish speaking Hispanic respondents were less likely to receive influenza vaccinations than non-Hispanic white respondents. These researchers concluded that ethnic disparities in the receipt of care were largely explained by differences in English fluency [23]. Fiscella and colleagues further reported that increasing the number of persons receiving influenza vaccination among Hispanics and other minority groups would significantly reduce the level of mortality among these groups [20]. Thus, our study provides additional evidence that Spanish language preference among older Hispanic persons may be an important barrier to receiving the influenza vaccine, which may adversely impact morbidity and mortality rates among the Hispanic population. And most recently, Dubard and Gizlice used data from earlier years of the same dataset, the BRFSS, to show decreased percentages of influenza vaccinations among Spanish-speaking Hispanic persons compared to English-speaking Hispanic persons [24]. Our study promotes this point even further by using more up to date data and using logistic regression models to take into account other variables which might affect influenza vaccination.

In addition to country-of-origin, length of time spent in the host country, and feelings of interaction with the new culture, language preference has been cited as one of the measures of “acculturation” [25, 26] which is defined as the cultural modification of an individual, group, or people by adapting to or borrowing traits from another culture. All of these measures can be associated with barriers to receiving care however, research has indicated that limited English proficiency remains a significant barrier to access and use of health care services among Hispanic persons, even when taking into consideration other measures of acculturation. Previous work has demonstrated that after adjusting for demographics, health status, and access to care variables, Hispanic persons with “fair” and “poor” English proficiency reported significantly less visits to their health care provider than did English speaking non-Hispanic persons [27]. In terms of acculturation, language preference is an important and measurable correlate to receiving adequate preventive health care services.

Several limitations should be considered when interpreting these analyses. First, the BRFSS is a land-line telephone survey, which excludes persons who have no telephone, and those with a cell phone only. Because of this, there is a possibility that some Hispanic households would not have had the opportunity to participate in this survey. However, because of the large sample size of the BRFSS, a relatively large sample of Hispanic persons was obtained, making this more likely to be representative of the Hispanic persons 65 years of age and older in the U.S.

A second limitation of this study is that determination of language preference was measured through the participants' choice of taking the survey in Spanish or English, so questions addressing cultural attitudes and beliefs about vaccines, as well as other measures of acculturation were not included. However, language preference only, as measured by preferred language for the survey or primary language spoken in the household, has been used in other studies to examine barriers to receiving adequate health care among Hispanic populations [11, 28, 29] as well as engagement in risky health behaviors [30, 31]. These studies have shown that language preference by itself is associated with negative health outcomes.

A third limitation of this study is that the cross-sectional nature of the data does not permit causal inferences in the observed associations to be made. However, the findings of the association between Spanish language preference and decreased receipt of influenza vaccinations were present and significant for three consecutive years.

A fourth limitation of this study was the self-report of influenza vaccination which is susceptible to recall bias. Although, findings from a study comparing several national surveys collecting health information, including receipt of influenza vaccinations, via different modes (i.e., in person and telephone) showed that responses to self-reported influenza vaccination had relatively small variation in reported rates across the different surveys [32]. By demonstrating similar response rates across surveys utilizing different modes of data collection, the validity of the information collected in this study is strengthened.

In conclusion, we found that among a large population-based sample of Hispanic persons aged 65 years and older, residing in the U.S., Spanish language preference was associated with lower rates of influenza vaccinations. The importance of this research is two-fold. Firstly, influenza infections can be more often fatal in elderly persons, and secondly, influenza may disparately affect minority populations such as Hispanic persons. Therefore, understanding barriers to receipt of an effective preventive health measure (e.g., vaccinations) is needed. Also, further research is needed to understand cultural sensitivities among Hispanic persons with regards to beliefs about vaccines in general.Such studies may provide further information about other barriers encountered by Hispanic persons with respect to receipt of influenza vaccinations. Information such as this may help increase vaccination rates and improve the overall well-being of older Hispanic persons in the United States. 

## Figures and Tables

**Table 1:** Population demographics and language preference for self-identified Hispanic persons, 65 years of age and older in the United States by year, 2005–2007.*

	2005		2006		2007	
	English	Spanish	English	Spanish	English	Spanish
Un-weighted sample size	1743	1587	1953	1996	2854	2271
Weighted sample size	1314000	1382000	1422000	1215000	1551000	1235000
Average age in years	73.6, (72.8–74.4)	72.8, (72.2–73.4)	73.1, (72.5–73.7)	73.4, (72.6–74.2)	72.9, (72.3–73.5)	73.1, (72.5–73.7)
% female	54.7 (48.7–60.6)	57.4 (51.6–63.0)	57.6 (52.2–62.8)	59.3 (53.7–64.6)	54.0 (49.3–58.6)	60.3 (55.3–65.0)
% completing high school or greater	67.2 (61.4–72.6)	32.4 (27.6–37.6)	67.9 (62.4–72.9)	35.3 (30.7–40.2)	72.9 (69.1–76.5)	37.6 (33.3–42.1)
% with any health care coverage	96.4 (93.0–98.2)	91.4 (88.2–93.8)	97.8 (96.3–98.6)	93.3 (89.8–95.6)	96.1 (94.6–97.2)	93.0 (90.6–94.8)
% with at least one health care provider	82.2 (78.0–85.7)	73.0 (68.4–77.2)	76.8 (71.9–81.1)	77.4 (73.0–81.3)	77.3 (73.1–81.0)	77.4 (73.8–80.7)

Source: Behavioral Risk Factor Surveillance System

*Estimates, (95% confidence intervals).

**Table 2:** Percentage of self-identified Hispanic persons aged 65 years and older receiving influenza vaccinations by language preference, 2005–2007.*

	2005	2006	2007
	*n* = 3330	*n* = 3949	*n* = 5125
English	55.8, (49.8–61.6)	60.3, (55.1–65.4)	63.4, (59.1–67.5)
Spanish	44.5, (39.0–50.2)	45.0, (39.4–50.6)	46.0, (41.1–50.9)
*P*-value**	<.01	<.01	<.01

Source: Behavioral Risk Factor Surveillance System

*Estimates, (95% confidence intervals)

**Chi-square test.

**Table 3:** Adjusted logistic regression modeling the odds of receiving recommended influenza vaccinations for self-identified Hispanic persons aged 65 years and older and choosing Spanish for a health interview in the United States, 2005–2007.*

	2005	2006	2007
	*n* = 3330	*n* = 3949	*n* = 5125
Language	Odds Ratio, (95% C.I.)	Odds Ratio, (95% C.I.)	Odds Ratio, (95% C.I.)
Spanish	0.67, (0.48–0.96)	0.53, (0.38–0.72)	0.50, (0.38–0.65)
English	reference	reference	reference

Source: Behavioral Risk Factor Surveillance System

*Logistic regression model controlling for age, sex, education, and health care coverage and having a personal health care provider.
